# A Comparative Study of Oral Health Status between International and Japanese University Student Patients in Japan

**DOI:** 10.3390/healthcare6020052

**Published:** 2018-05-22

**Authors:** Ai Ohsato, Masanobu Abe, Kazumi Ohkubo, Hidemi Yoshimasu, Liang Zong, Kazuto Hoshi, Tsuyoshi Takato, Shintaro Yanagimoto, Kazuhiko Yamamoto

**Affiliations:** 1 Division for Health Service Promotion, the University of Tokyo, 7-3-1 Hongo, Bunkyo-ku, Tokyo 113-0033, Japan; osato.ai@mail.u-tokyo.ac.jp (A.O.); yanagimoto@hc.u-tokyo.ac.jp (S.Y.); yamamoto-tky@umin.ac.jp (K.Y.); 2 School of Oral Health Sciences, Faculty of Dentistry, Tokyo Medical and Dental University, Tokyo 113-8549, Japan; h-yoshimasu.ocsh@tmd.ac.jp or phyoshimasu@outlook.jp; 3 Oral and Maxillofacial Surgery, Dentistry and Orthodontics, the University of Tokyo Hospital, Tokyo 113-8655, Japan; okubok-ora@h.u-tokyo.ac.jp (K.O.); hoshi-ora@h.u-tokyo.ac.jp (K.H.); t-takato@jreast.co.jp (T.T.); 4 Graduate School of Medicine, the University of Tokyo, Tokyo 113-8655, Japan; zl20014111@163.com; 5 Key Laboratory of Carcinogenesis and Translational Research (Ministry of Education), Department of Gastrointestinal Surgery, Peking University Cancer Hospital & Institute, Beijing 100142, China

**Keywords:** oral health, university student, check-up, DMFT

## Abstract

*Background:* The number of international students enrolled in universities in Japan is increasing. To provide better oral care services for international students, we have to understand their oral environment and dental health behaviors. However, few studies have investigated the oral health status of international university students. The object of the present study was to clarify the current oral status of international university students. *Methods:* The subjects were students who visited the dental department at the University of Tokyo’s Health Services Center between April 2012 and March 2013. Our medical records were reviewed with regard to the following items: attributes (nationality, gender, and age); chief complaint (reason for visit); history of dental treatment; mean number of decayed (D), missing (M) or filled (F) teeth as a single (DMFT) index; degree of calculus deposition; gingival condition; and oral hygiene status. *Results:* The records of 554 university students (138 international and 416 non-international students) were analyzed; 88.4% of the 138 international students were from Asian countries (*n* = 122), of which 47.1% were from China and 10.9% from Korea, followed by North America (5.8%), Europe (4.3%), and Africa (1.5%). Although no significant differences were found regarding the history of dental treatment between international and non-international students (49.3% and 48.8%, respectively), international students had a significantly higher dental caries morbidity rate (60.1%) than non-international students (49.0%). The international students showed a significantly higher DMFT value compared with the non-international students: 5.0 and 4.0 per individual, respectively. Severe calculus deposition was observed in international students compared with non-international students (51.9% and 31.7%, respectively). *Conclusions:* The international university students had poorer oral health status than the non-international students, even though the result might include many uncertainties and possible biases.

## 1. Introduction

The present study was conducted to clarify the oral environment and dental health behaviors of university students who visited the dental clinic at the University of Tokyo’s Health Services Center. Oral diseases, particularly periodontal disease, are evidently the leading cause of tooth loss in adolescence and subsequent life stages [1]. Current evidence suggests that poor oral health status influences systemic disorders, such as cardiovascular disease, diabetes mellitus, etc. [2,3,4,5,6,7]. Therefore, strengthening regular oral check-ups for young people is considered necessary to prevent not only oral diseases but also systemic diseases [8,9,10].

According to the Japan Student Services Organization (JASSO), an independent administrative institution under the aegis of the Japanese Ministry of Education, Culture, Sports, Science and Technology (MEXT), the number of international university students in Japan has increased by 18% in the past 5 years, with students from Asian countries comprising the largest portion of the international student population. To provide better oral care services for international students, we have to understand the features of their oral health status, which might be affected by many social factors such as cultural differences, health insurance system differences, dental access differences, etc. However, few studies have compared the oral status between international and non-international university students [11]. 

The present study was conducted to clarify the oral environment and dental health behaviors of international university students who visited the dental clinic in Health Services Center in University. 

## 2. Methods

### 2.1. Ethics Approval and Consent to Participate

This research was approved by Institutional Review Board of the University of Tokyo (the approval number 13-146). Because this was a retrospective observational study and included no therapeutic intervention, the need for informed consent has been waived by the Institutional Review Board.

### 2.2. Subjects

Our subjects consisted of 554 students (138 international students and 416 non-international students) who visited the dental department at the university’s Health Service Center between April 2012 and March 2013. All accessible data were collected. Students who held Japanese nationality (*n* = 414) or who were permanent residents of Japan (*n* = 2) were classified into the group of non-international students. Among 138 international students, 60.1% and 39.9% were male and female, and among the 416 non-international students, 74.5% and 25.5% were male and female, respectively. We reviewed the students’ medical records available at the Health Service Center with regard to the following items: attributes (nationality, gender, and age), chief complaint (reason for visit), history of dental treatment, and mean number of decayed, missing or filled teeth in a single (DMFT) index, degree of calculus deposition and oral hygiene status. Calculus grading scale was as follows, NO: No calculus; MILD: calculus deposits less than one second of the tooth surface; SEVERE: Calculus deposits greater than one second of the tooth surface and/or extends subgingival. Categories of oral hygiene status was as follows, GOOD: No or slight dental plaque deposits on the several teeth; POOR: heavy dental plaque deposits extending to all the teeth.

### 2.3. Analytical Procedures

The retrospectively obtained survey results were anonymized from the original dental charts and converted to numeric data, which were statistically analyzed using the chi-square test, question-specific simple tabulation, and cross tabulation. As a rule, the level of significance was set at *p* < 0.05.

## 3. Results

**Figure 1:**
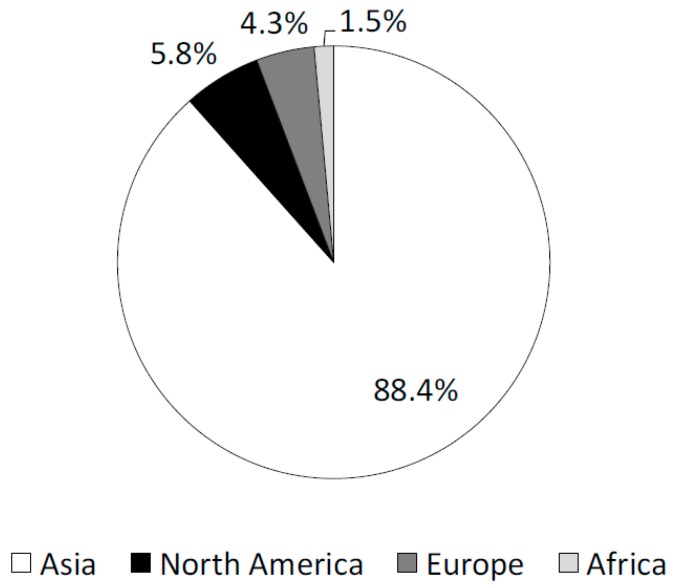
Home countries of the international students visiting our university dental clinic. The most common origin of the international students was Asia, followed by North America, Europe, and Africa.

The mean age was 28 ± 3 years for the 138 international students and 25 ± 4 years for the 416 non-international students, with an overall mean age of 26 ± 4 years. The international students tended to be older than the non-international students, but the difference was not significant. The most common home origin of the international students was Asia, accounting for 88.4%, followed by North America (5.8%), Europe (4.3%), and Africa (1.5%). Of the students from Asia the percentages from China and Korea were particularly high (47.1% and 10.9%, respectively) (Figure 1). 

Our subject classification by chief complaint (reason for visit) showed that 48.6% of the non-international students wanted a dental checkup, 20.8% wanted dental cleaning, 17.1% wanted dental caries treatment, and 8.6% wanted a dental consultation. Among the international students, 30.1% wanted a dental checkup, 28.2% wanted dental caries treatment, 19.9% wanted a dental consultation, and 17.9% wanted dental cleaning. A higher percentage of international students complained of specific oral symptoms compared to the non-international students (Supplementary Figure S1).

**Figure 2:**
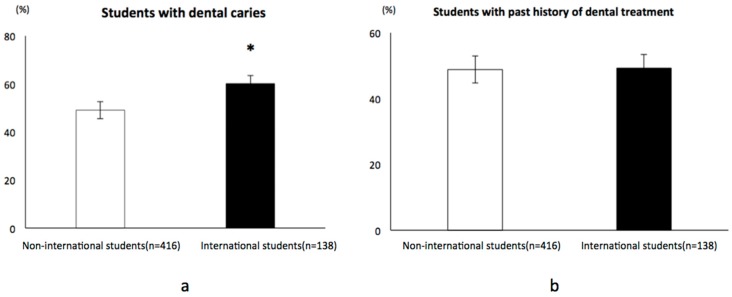
Comparison of morbidity of dental caries, past history of tooth extraction and dental treatment between international and non-international students in Japan. (**a**) Non-international students had a significantly lower dental caries morbidity rate than the international students (*p* < 0.05); * *p* < 0.05. (**b**) The rate of dental treatment was similar between international and non-international students in Japan.

Dental caries occurred at significantly higher rates in the international students than in non-international students (*p* < 0.05). The dental caries morbidity rates were 60.1% and 49.0% in international and non-international students, respectively. However, the rate of past dental treatment was similar between international and non-international students: 49.3% and 48.8%, respectively (Figure 2). 

**Figure 3:**
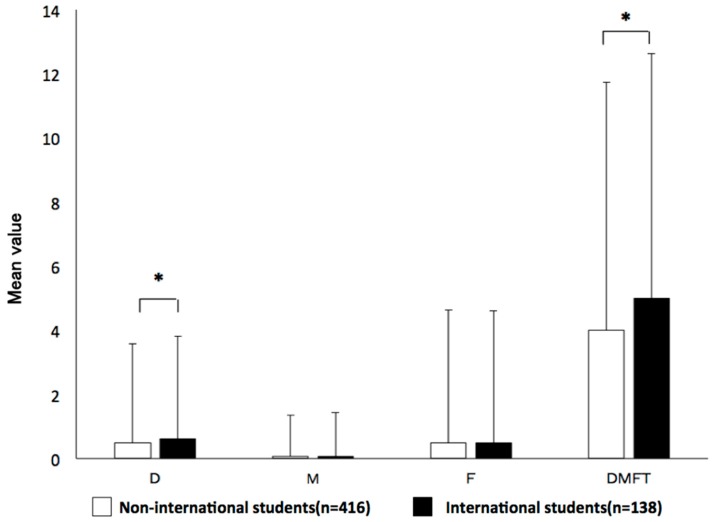
Comparison of the decayed, missing or filled teeth (DMFT) index between international and non-international students. Compared with the international students, the non-international students had a significantly lower value of DMFT, lower number of decayed teeth (D). Missing teeth (M) and filled teeth (F) showed no significant differences between international and non-international students in Japan. * *p* < 0.05.

Compared with the international students, the non-international students had significantly lower mean numbers of DMFT (4.0 vs. 5.0 per individual; *p* < 0.05) and decayed teeth (D) (1.8 vs. 2.2; *p* < 0.05). Missing teeth (M) and filled teeth (F) showed no significant differences between international and non-international students (Figure 3).

**Figure 4:**
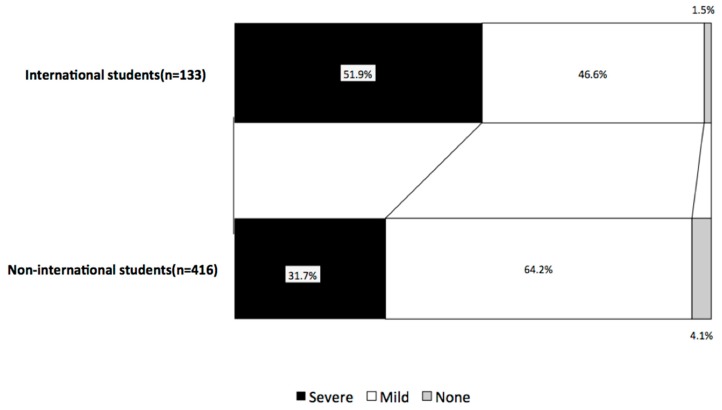
Comparison of the calculus deposition by severity. The calculus deposition was more severe in the international students than the non-international students in Japan.

The calculus deposition was severe in 51.9% of the international students and in 31.7% of the non-international students. The international students were significantly more likely than the non-international students to have severe dental conditions (Figure 4).

The dental hygiene status at the first visit was rated good or average for 40.9% for the international students and 66.2% of the non-international students. The percentage of international students with poor dental hygiene status was 59.0%; that of the non-international students was 33.8% (*p* < 0.05, Supplementary Figure S2).

## 4. Discussion

The number of foreign university students in Japan increased by 18% in the past 5 years. Enhanced promotion of dental hygiene practices is necessary for foreign university students. The number of international university students is increasing year by year in Japan. The total number of university students at the University of Tokyo was 56,319, of which only 554 visited the dental department at the university’s Health Services Center from April 2012 to March 2013. Of these 554 students, 138 (24.9%) were from foreign countries. The large fraction of international university students who visited the department of dentistry had poorer dental health status than non-international students, although the current result might include a selection bias because our target is “student patients”, not “students”. We analyzed “student patients” instead of “students” in this study. Therefore, the validation of the data in another population of “students” is necessary. The majority of the international students were from Asia (88.4%); in particular, students from China accounted for a large fraction of the international students (47.1%). The comparative investigation between international students from Asia and non-international students was performed. The outcomes of analyses were not largely changed even if the students from North America, Europe and Africa were excluded (data was not shown).

The ratio of people to dentists in China is much lower (10,000:1.1) than that in Japan (10,000:7.1) (Working Together for Health—The World Health Report 2006, WHO). This might mean that people have less chance to access adequate treatment from the dentists in China [12]. This could be one of the reasons for the differences in oral health status we found. The difference of the programs of health insurance coverage between China and Japan (affordability of dental care) might be also important. There are two major types of insurance programs available in Japan: Employee Health Insurance and National Health Insurance, and both programs cover basic dental treatments. 

On the other hand, Chu et al. emphasized the importance of oral health education [8]. They pointed out that the level of awareness and knowledge about dental erosion is generally low in Chinese urban areas even though most such residents receive regular dental check-ups. Ohshima et al. compared periodontal health status and oral health behavior between Japanese and Chinese dental students, and found that periodontal disease rates were much higher in Chinese students than Japanese students, even though dental students may not represent the general university population [11]. They concluded that this might result from different lifestyles and oral hygiene habits. Peltzer et al. investigated the oral health behavior of university students in 26 countries and concluded that the behavior was affected by the social and health factors [13]. In our present study, although international students showed poorer oral health status than the non-international students, there was no significant difference between the two groups in the history of dental treatment. The previous studies indicate the importance of awareness and knowledge of oral health issues. Accordingly, the establishment and the strengthening of oral hygiene education are urgently needed to prevent periodontal and dental diseases [8,9,10].

Early detection of life-threatening diseases is considered a preferential issue, with dental care considered of lower priority. However, emerging evidence suggests that poor oral health influences not only oral diseases such as dental caries and periodontitis but also systemic disorders such as cardiovascular disease, diabetes mellitus, rheumatoid arthritis, Alzheimer’s disease, metabolic syndrome, preterm delivery, and osteoporosis, to name a few [2,3,4,5,6,7]. We have also shown that the imbalance of oral bacterial flora deriving from poor oral health can lead to several systemic diseases [14,15,16,17,18,19]. Therefore, maintaining good oral health is crucial for not only prevention of local oral diseases but also prevention and management of systemic diseases [20]. 

To prevent oral and systemic diseases, the most important step is encouraging students to have regular check-ups and cleanings. In addition, improving self-efficacy has been reported to be beneficial for maintaining good oral health in university students [21]. The number of international university students in Japan increased by 18% in the past 5 years. Enhanced promotion of dental hygiene practices is necessary for international university students.

## 5. Conclusions

International students presented poorer oral health statuses than non-international students in Japan, even though the result might include many uncertainties and possible biases. Encouraging regular oral check-ups and cleanings and education for self-efficacy are necessary to improve oral health and prevent systemic diseases.

## Supplementary Materials

The following are available online at http://www.mdpi.com/2227-9032/6/2/52/s1, Figure S1: State of Oral Hygiene shows the variation in complaints of specific oral symptoms between international and non-international university students, Figure S2: Comparison of oral hygiene status. The percentage of international students with poor dental hygiene status was higher than that of the non-international students in Japan.
