# Supplementary material for: A Comparative Study of Oral Health Status between International and Japanese University Student Patients in Japan
Source: Healthcare (Basel). 2018 May 22;6(2):52. doi: 10.3390/healthcare6020052 (PMC6023454; doi:10.3390/healthcare6020052)
Supplement: Supplementary file 1 [file healthcare-06-00052-s001.pdf]

Supplementary Materials: A Comparative Study of Oral Health Status between International and Japanese University Student Patients in Japan

Ai Ohsato, Masanobu Abe, Kazumi Ohkubo, Hidemi Yoshimasu, Liang Zong, Kazuto Hoshi, Tsuyoshi Takato, Shintaro Yanagimoto and Kazuhiko Yamamoto

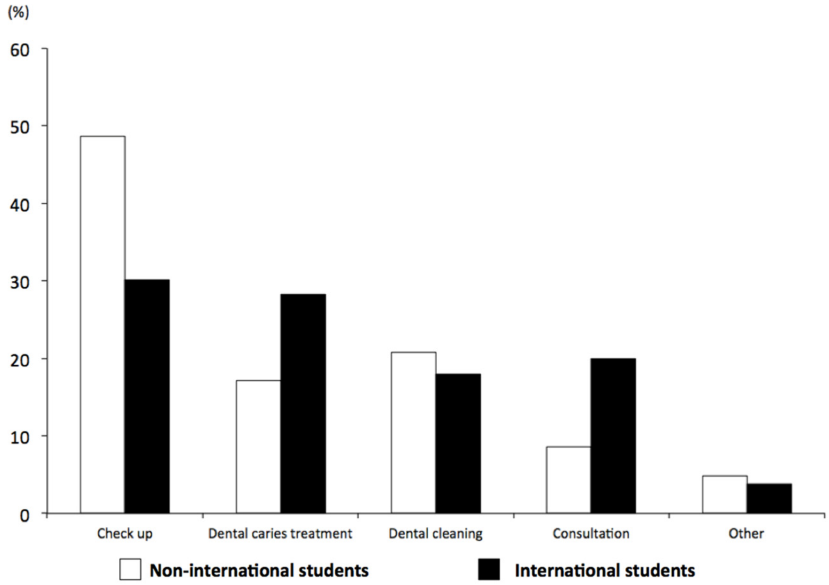

Figure S1. State of Oral Hygiene shows the variation in complaints of specific oral symptoms between international and non-international university students.

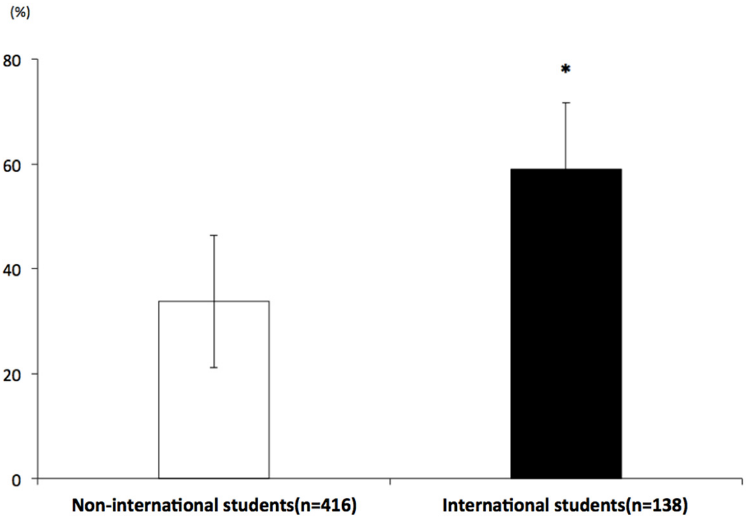

Figure S2. Comparison of oral hygiene status. The percentage of international students with poor dental hygiene status was higher than that of the non-international students in Japan. *$p<0.05$.

© 2018 by the authors. Licensee MDPI, Basel, Switzerland. This article is an open access article distributed under the terms and conditions of the Creative Commons Attribution (CC BY) license (http://creativecommons.org/licenses/by/4.0/).
